# Massive-mode polarization entangled biphoton frequency comb

**DOI:** 10.1038/s41598-022-12691-7

**Published:** 2022-05-27

**Authors:** Tomohiro Yamazaki, Rikizo Ikuta, Toshiki Kobayashi, Shigehito Miki, Fumihiro China, Hirotaka Terai, Nobuyuki Imoto, Takashi Yamamoto

**Affiliations:** 1 Graduate School of Engineering Science, Osaka University, Toyonaka, Osaka 560-8531 Japan; 2 Center for Quantum Information and Quantum Biology, Osaka University, Osaka, 560-8531 Japan; 3 Advanced ICT Research Institute, National Institute of Information and Communications Technology (NICT), Kobe, 651-2492 Japan; 4 Graduate School of Engineering, Kobe University, Kobe, 657-0013 Japan

**Keywords:** Quantum information, Single photons and quantum effects, Quantum optics, Single photons and quantum effects, Nonlinear optics, Frequency combs

## Abstract

A frequency-multiplexed entangled photon pair and a high-dimensional hyperentangled photon pair are useful to realize a high-capacity quantum communication. A biphoton frequency comb (BFC) with entanglement can be used to prepare both states. We demonstrate polarization entangled BFCs with over 1400 frequency modes, which is approximately two orders of magnitude larger than those of earlier entangled BFCs, by placing a singly resonant periodically poled LiNbO_3_ waveguide resonator within a Sagnac loop. The BFCs are demonstrated by measuring the joint spectral intensity, cross-correlation, and autocorrelation. Moreover, the polarization entanglement at representative groups of frequency modes is verified by quantum state tomography, where each fidelity is over 0.7. The efficient generation of a massive-mode entangled BFC is expected to accelerate the increase of capacity in quantum communication.

## Introduction

Entangled photon pairs are essential resources for quantum information processing such as quantum communication^1,2^, quantum computation^3,4^, and quantum metrology^5^. Fully utilizing the degrees of freedom (DOFs) of photons remain a crucial challenge in developing better photon resources. Recently, the quantum nature of the frequency DOF of photons has been studied intensively^6,7^. To this end, a quantum frequency comb^8,9^, which is the superposition of different frequency modes at a certain mode spacing, is gaining research interest owing to its advantage in the generation^10–12^ and manipulation^13–16^ of high-dimensional quantum states. A quantum frequency comb formed by a frequency-entangled photon pair, i.e. a biphoton frequency comb (BFC), is a building block of quantum information processing that relies on its frequency DOF.

BFCs are generated efficiently via spontaneous parametric down-conversion (SPDC) or spontaneous four-wave mixing (SFWM) inside a cavity. On the one hand, a photon pair generated by SPDC/SFWM without a cavity has a broad bandwidth, which can be determined by the phase-matching condition. On the other hand, photon pair generation in SPDC/SFWM inside a cavity occurs intensively in the resonant frequency modes of the cavity; this results in the highly efficient generation of BFCs. Thus far, BFCs have mostly been generated using SFWM^7,10,17–19^ and SPDC^20,21^ with micro-ring resonators^22^. However, the number of their frequency modes is limited to a few dozen because of the smallness of the cavity size and “the cluster effect”, which is the suppression of photon pair generation in a certain frequency range, in a doubly resonant configuration^23,24^. In our experiment, we use a monolithic periodically poled LiNbO_3_ integrated waveguide resonator (PPLN/WR). Its cavity confines only the longer-wavelength (signal) photons of the photon pair, and it does not confine shorter-wavelength (idler) photons to avoid the cluster effect^11^; thus, a BFC with over 1400 frequency modes is achieved. We demonstrate the generation of polarization entangled BFC ranging over 1400 modes, which is about two orders of magnitude larger than previous entangled BFCs, by placing the singly resonant PPLN/WR into a Sagnac interferometer. In the Sagnac interferometer, the BFCs for horizontal and vertical polarizations are generated by the same PPLN/WR, and therefore, a perfect spectral overlap between the H- and V-polarized BFCs can be ensured.

Additional entanglement in another DOF of the BFC extends the use case to various applications such as (a) high-dimensional hyperentangled photon pairs^15,25,26^ or (b) frequency-multiplexed entangled photon pairs^18^. Suppose a simplified model for generating polarization entangled BFCs based on SPDC, where the generated state is written by

1 $$\begin{aligned} |\Psi \rangle = \left( \prod _{m=1}^M S_{m,H}(\zeta ) S_{m,V}(\zeta )\right) |0\rangle . \end{aligned}$$

$S_{m,H(V)}(\zeta )$ represents a two-mode squeezing operator where $m~(1\le m\le M)$ and H(V) denote the frequency mode index and the horizontal (vertical) polarization, respectively. The complex squeezing parameter $\zeta$ is proportional to the complex amplitude of the SPDC pump light.

From Eq. (1), we prepare two different states (a) and (b) by adjusting only the pump power through parameter $\zeta$ as follows: (i) In the low pump power regime satisfying $2M |\zeta |^2 \ll 1$, a single photon pair state is represented by

2 $$\begin{aligned} |\Psi \rangle \simeq \frac{1}{\sqrt{2}} (|HH\rangle _{i,s}+|VV\rangle _{i,s})\otimes \frac{1}{\sqrt{M}} \left( \sum _{m=1}^M |\omega _{m} \omega _{m}\rangle _{i,s}\right) , \end{aligned}$$

where $|\omega _{i(s),m}\rangle$ represents the state of the idler (signal) photon in the frequency index *m*. This state corresponds to a high-dimensional hyperentangled state (a) of polarization and frequency, which is valuable for many protocols with hyperentanglement ^27–30^. (ii) For $2 |\zeta |^2 \ll 1$, the generated state in each frequency mode is approximately a single photon pair, and it is represented as

3 $$\begin{aligned} |\Psi \rangle \propto \bigotimes _{m=1}^M \left[ |0\rangle _m+\zeta \left( |HH\rangle _{{i,m},{s,m}}+|VV\rangle _{{i,m},{s,m}}\right) \right] . \end{aligned}$$

This state is equivalent to the state formed by many polarization-entangled photon pairs with different frequency indices that share a single pump light. This frequency-multiplexed entangled photon pair (b) can be applied to broadband entanglement-distribution network^31–36^. Conditions of the pump power of (a) and (b) are different by *M*, which becomes remarkable in a massive mode BFC. In our experiment, we adjust the pump power based on the frequency window of the measurements. A realistic model for polarization-entangled BFCs is discussed in the Supplementary Information.

## Results

**Figure 1:**
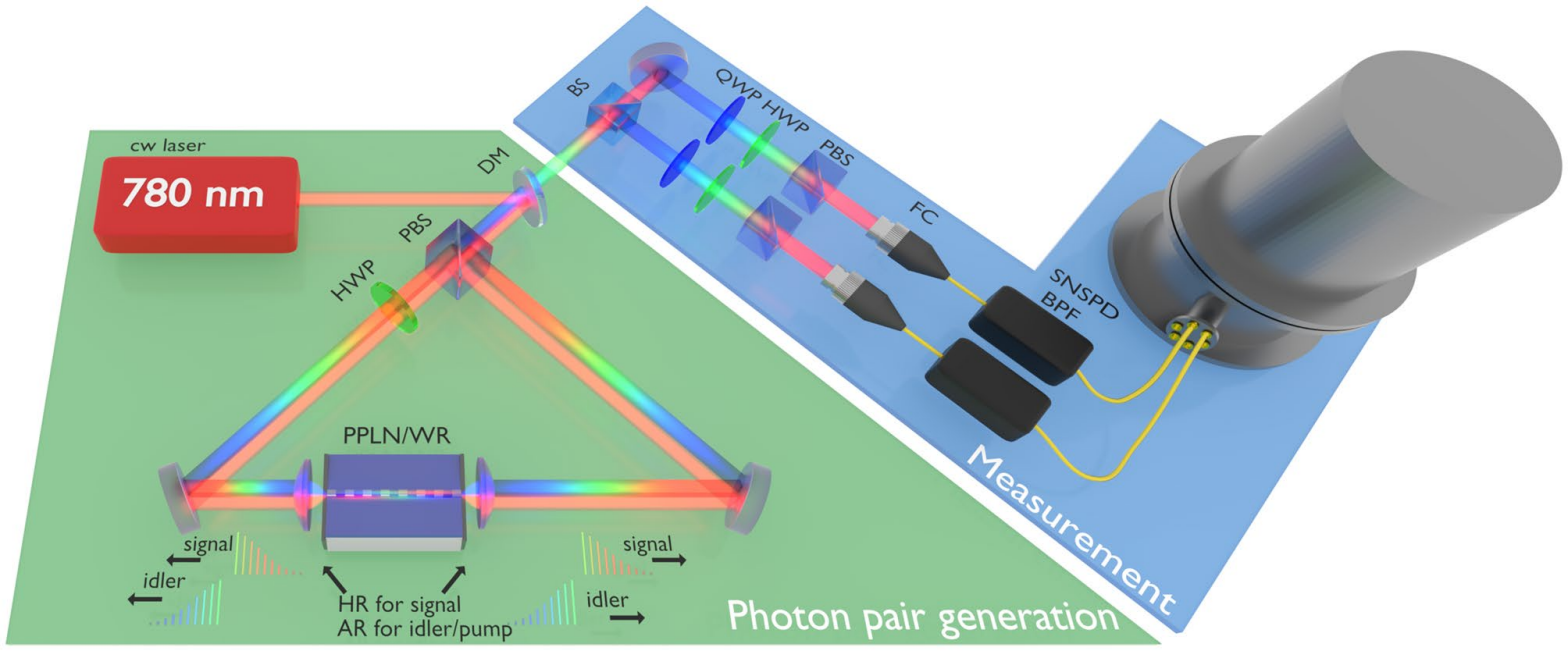
Experimental setup for the generation and measurement of quantum states. *BS* beam splitter, *PBS* polarizing beam splitter, *HWP* half-wave plate, *QWP* quarter-wave plate, *DM* dichroic mirror, *FC* fiber coupler, *BPF* bandpass filter, *SNSPD* superconducting nanowire single-photon detector, *PPLN/WR* periodically poled LiNbO_3_ waveguide resonator.

Figure 1 shows the experimental setup. We used a continuous-wave (CW) pump laser at 780.24 nm, whose power is adjustable from 50 μW to 5 mW. The Sagnac interferometer comprises a polarizing beam splitter (PBS), PPLN/WR, and half-wave plate (HWP). In the Sagnac interferometer, $|HH\rangle$ and $|VV\rangle$ photon pairs are generated in the clockwise and anticlockwise directions, respectively. The photon pairs are mixed by PBS and propagated in the direction opposite to that of the pump laser. The generated quantum state is a polarization-entangled BFC, which corresponds to Eq. (1). The setup does not require any stabilization besides the temperature control of the PPLN/WR because of the monolithic structure of PPLN/WR and the phase stability of a Sagnac interferometer^37,38^.

The two photons are divided using a 50:50 beam splitter (BS) to evaluate the quantum states. Each photon goes to the setup for polarization quantum state tomography with a tunable bandpass filter (BPF). Then, they are detected using superconducting nanowire single-photon detectors (SNSPDs)^39^. In the Sagnac interferometer, the undesired photon pairs $|HV\rangle$ and $|VH\rangle$ are generated by the signal components reflected at the resonator. We postselect only the desired photon pairs composed of the idler photons and the transmitted signal photons by time-resolved coincidence measurements by placing the PPLN/WR off-center from the interferometer (see Supplementary Information for details).

**Figure 2:**
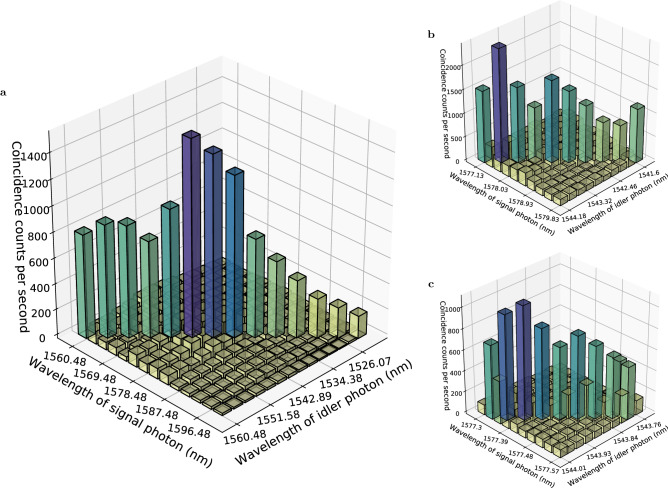
Joint spectral intensity. Coincidence counts among frequency modes with spectral resolutions of (**a**) 3.00 nm, (**b**) 0.30 nm, and (**c**) 0.03 nm are shown. The resolutions are selected using the BPFs. The pump powers are adjusted based on the resolution to 50 μW, 500 μW, and 5 mW for (**a**–**c**), respectively. Peaks appear at the specific frequency combinations that satisfy the energy conservation with the pump, signal, and idler photons in all frequency ranges measured: (**a**) [1560.48 nm, 1599.48 nm] and [1522.43 nm, 1560.48 nm], (**b**) [1577.13 nm, 1579.83 nm] and [1541.60 nm, 1544.18 nm], and (**c**) [1577.30 nm, 1577.57 nm] and [1543.76 nm, 1544.01 nm]. The range of (**b**) is equivalent to the spectral resolution of (**a**) and the range of (**c**) is the spectral resolution of (**b**).

To observe the frequency-correlated photon pair generation over a wide spectral range, we first measure the joint spectral intensity (JSI) over 80 nm (1520–1600 nm) with a resolution of 3.00 nm, as shown in Fig. 2a. The JSIs with higher resolutions (0.30 nm and 0.03 nm) are shown in Fig. 2b,c, respectively. The peaks of the coincidence counts clearly obey the energy conservation and exhibit a frequency correlation in all frequency ranges. The non-uniform shape of the peaks is qualitatively explained by the slightly remaining cluster effect attributed to the imperfection of the singly resonant configuration around the degenerate point of 1560.48 nm, as indicated in Fig. S1f in the Supplementary Information.

**Figure 3:**
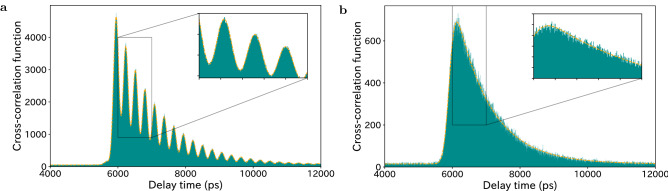
Temporal second-order cross-correlation. The delayed coincidence counts between the signal and idler photons with 4-ps time resolution, which correspond to the temporal second-order cross-correlation function. The polarizations are fixed in the horizontal direction, and the wavelengths of the signal and idler photons are 1580.48 nm and 1540.98 nm, respectively. The bandwidths of the BPFs are (**a**) 3.00 nm ($\sim$ 100 frequency mode) and (**b**) 0.03 nm (single-frequency mode). The data recording time is 1000 s. In (**a**), we observe the beating of coincidence counts, which is significant evidence of the quantum frequency comb.

The temporal second-order cross-correlation between the signal and idler photons illustrates an additional spectral property of the quantum frequency comb. Figure 3 shows an example of the results for the central wavelengths of 1580.48 nm and 1540.98 nm (see Supplementary Information for other frequency ranges). The cross-correlation with a 3.00 nm filter bandwidth in Fig. 3a shows a beat signal reflecting the superposition of multiple frequency modes. A beat frequency of 3.5 GHz corresponds to the free spectral range (FSR) of the cavity. The beat signal in the delayed coincidence counts disappears when a single frequency mode is picked up by the filters with a 0.03 nm bandwidth corresponding to 3.5–3.7 GHz, as shown in Fig. 3b. We estimate the full width at half maximum (FWHM) of the linewidth of the frequency mode at approximately 1580 nm to be $\Delta f_{1/2}=126$ MHz based on the decay time of the cross-correlation. This implies that the comb teeth are well separated from each other because of the strong confinement of the signal photons in the resonator. In addition to the results shown in Fig. 2, wherein the photon pairs appear over 80 nm (1520–1600 nm), the clear beat signal implies the existence of frequency entanglement in the BFC with a frequency mode number of $M\sim 1400$.

**Figure 4:**
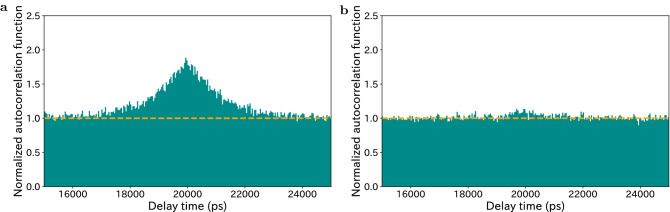
Temporal second-order autocorrelation. The delayed coincidence counts between idler photons with 40-ps time resolution; this corresponds to the temporal second-order autocorrelation function. The polarizations are fixed in the horizontal direction. The central wavelength and bandwidth of the BPFs are respectively set to (**a**) 1543.76 nm and 0.03 nm and (**b**) 1543.89 nm and 0.30 nm. The data recording time is 1000 s. The orange lines represent the normalization factors estimated from the coincidence counts with a large delay. In (**a**), the bunching effect appears at the zero delay because unheralded photons behave like thermal light in the single-frequency mode. However, in (**b**), it is suppressed by the interference in multiple frequency modes.

The mode number contained in the dense and well-separated quantum frequency comb can be estimated using the temporal second-order autocorrelation function $g^{(2)}(\tau )$ of the signal (idler) photons. This method is well established in the pulsed pump SPDC, but not in the CW pump case. We derive the relation $\Delta g^{(2)} = (\pi \Delta f_{1/2}M)^{-1}$ for the CW-pumped SPDC with a singly resonant cavity by defining the time-integrated autocorrelation by $\Delta g^{(2)} = \int d\tau \big (g^{(2)}(\tau )-1\big )$; it is similar to the relation between $g^{(2)}$ and the effective mode (Schmidt mode) for the pulsed-pump regime^40^. We obtain $\Delta g^{(2)} = 2.1$ and 0.17 ns from the observed $g^{(2)}(\tau )$ with bandwidths of 0.03 nm and 0.30 nm, respectively, as shown in Fig. 4a,b. We estimated *M* to be 1.2 and 14.9 for bandwidths of 0.03 nm and 0.30 nm, respectively, by using $\Delta f_{1/2}=126$ MHz. This is consistent with the mode numbers 1 and 10 expected from the beat frequency.

**Figure 5:**
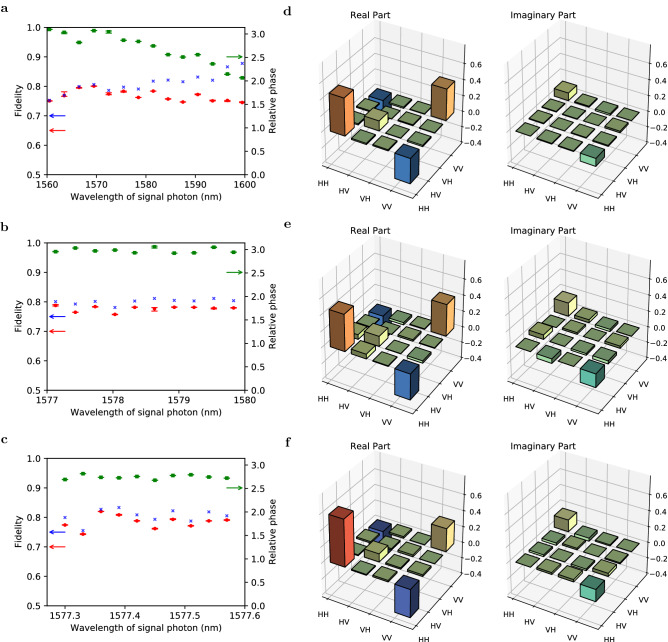
Fidelities and reconstructed density matrices of polarization entangled states. We performed a set of polarization measurements with various central wavelengths and bandwidths of BPFs. The data recording time at each polarization setting is 200 s. (**a**–**c**) Red, green, and blue markers represent the fidelity of the quantum state, relative phases between the polarizations of HH and VV of the reconstructed states, and expected fidelity after removing contamination with undesired components, respectively (see Supplementary Information). The error bars represent standard deviations under the assumption of Poisson statistics for the photon counts. The frequency ranges are (**a**) [1560.48 nm, 1599.48 nm] and [1522.43 nm, 1560.48 nm], (**b**) [1577.13 nm, 1579.83 nm] and [1541.60 nm, 1544.18 nm], and (**c**) [1577.30 nm, 1577.57 nm] and [1543.76 nm, 1544.01 nm] for bandwidths of 3.00 nm, 0.30 nm, and 0.03 nm, which correspond to $\sim$ 100, 10, and 1 frequency modes, respectively. Examples of the reconstructed density matrix are shown in (**d**–**f**) with spectral resolutions of 3.00 nm, 0.30 nm, and 0.03 nm, respectively. The combinations of the frequency modes are (**d**) 1578.48 nm and 1542.89 nm, (**e**) 1577.43 nm and 1543.89 nm, and (**f**) 1577.39 nm and 1543.93 nm.

Finally, we perform the quantum state tomography of the polarization DOF^41^ at various central frequencies. We adjust the pump power to 50 μW, 500 μW, and 5 mW for filter bandwidths of 3.00 nm, 0.30 nm, and 0.03 nm, respectively, where the excitation rates over a coincidence time window of 1100 ps are below 0.01. Examples of the matrix representations of the reconstructed density operators $\rho$ are shown in Fig. 5d–f. Fidelity is defined by $F(\rho ) = \max _\theta \langle\Psi _\theta| \rho | \Psi _\theta \rangle$, where $|\Psi _\theta \rangle = \big (|H\rangle |H\rangle +e^{i\theta } |V\rangle |V\rangle \big )/\sqrt{2}$ is a maximally entangled state with a relative phase $\theta$. Figure 5a–c show that the observed fidelities for all frequency regions and bandwidth settings are over 0.7, which clearly exceeds 0.5; this implies that quantum entanglement in the polarization subspace exists in all frequency ranges. See Fig. S5 in the Supplementary Information for other characterization of the generated states.

## Discussion

We now discuss how to improve the fidelity of the observed states. One of the reasons for the decrease in fidelity is contamination with undesired components $|HV\rangle$ and $|VH\rangle$ caused by the insufficient difference between the two path lengths on the Sagnac interferometer (Fig. S3 in the Supplementary Information). The contamination can be prevented by placing the PPLN/WR in a more off-centered position of the Sagnac interferometer. Another solution is to place the PPLN/WR in the centered position and stabilizing the interferometer, which can not only improve the fidelity but also double the generation rate. The expected fidelities after removing these contaminants are shown in Fig. 5a–c by blue markers. However, there remains a non-negligible decrease in the fidelity. Although this can be attributed to experimental imperfections such as the insufficient temperature control of the PPLN/WR, further investigations are required in the future.

We developed polarization-entangled BFCs with over 1000 frequency modes using a singly resonant PPLN/WR in a Sagnac interferometer. We showed that the SPDC-based entangled BFC generator enables us to prepare both high-dimensional hyperentangled photon pairs and frequency-multiplexed entangled photon pairs by adjusting the pump power for the SPDC. The millimeter-long cavity structure realizes a comb spacing from gigahertz to tens of gigahertz, and therefore, commercially available filters and wavelength-division multiplexing devices can help realize a broadband quantum communication network. In addition, the presented setup has the potential to integrate into one chip^42,43^. Thus, this versatile, stable, and highly efficient system for the generation of the massive-mode entangled BFCs can help provide a platform for high-capacity and highly efficient quantum information processing.

## Methods

### Singly resonant PPLN/WR

We used a 20 mm long ridge waveguide that consists of a zinc-doped lithium niobate as the core and lithium tantalite as a clad (NTT Electronics), as a nonlinear optical waveguide. The periodic-poling period was designed to satisfy the type-0 quasi-phase-matching of the second harmonic generation of 1560.48 nm light at 35 $^\circ$C. Both end-faces of the waveguide were flat polished for the Fabry–Pérot cavity structure and coated by dielectric multilayers for a highly reflective coating around 1600 nm and anti-reflective coatings around 1520 nm and 780 nm^11^. The waveguide resonator was stabilized by temperature control using a Peltier device.

### Experimental setup

The pump light is generated by second-harmonic generation in PPLN/W pumped by an external cavity laser with a planar lightwave circuit (RIO Planex) at 1560.48 nm. The power and polarization of the pump light were adjusted using a pair of HWPs sandwiched between a PBS. We set the power of the pump light to 50 μW, 500 μW, and 5 mW to maintain the effective $M\zeta$ for different bandwidths of the BPFs, 3.00 nm, 0.30 nm, and 0.03 nm, respectively.

A BFC generated at the Sagnac interferometer was separated from the pump light by a dichroic mirror (DM), and it was divided into two paths by a BS with a probability of 1/2. Each photon was projected onto any polarization state using an HWP, a quarter-wave plate(QWP), and a PBS. The photon was coupled to a single-mode fiber connected to a tunable BPF (Alnair Labs), whose bandwidth could be adjusted between 0.03 and 3.00 nm. Finally, the photon was detected using a SNSPD^39^. The electrical signals from the two SNSPDs were used as the start and stop of a time-to-digital converter (PicoQuant) to record the coincidence counts with timestamps. For quantum state tomography, the coincidence counts were totaled in the time windows of 5800–6900 ps.

### Formula for estimating the number of cavity modes in the CW pump regime

In the pulsed pump regime, the autocorrelation function of the signal or idler photons of photon pairs is related to the number of effective (Schmidt) modes by $g^{(2)} = 1+\frac{1}{N}$ under the assumption of a uniform spectral amplitude^40^. However, this formula cannot be applied directly to the CW-pumped regime. Autocorrelation is related to the cavity mode in the CW-pumped regime, which is the same as the effective mode only in the case of ideal frequency-bin entangled states. We obtained a formula

4 $$\begin{aligned} \Delta g^{(2)}&\equiv \int d\tau \bigg (g^{(2)} (\tau )-1\bigg ) \nonumber \\&=\frac{1}{M} \frac{\gamma _i^2+3\gamma _i\gamma _s+\gamma _s^2}{\gamma _i \gamma _s(\gamma _i+\gamma _s)} \nonumber \\& = {\left\{ \begin{array}{ll} \frac{5}{2\gamma _s M} &{} (\gamma _s=\gamma _i) \\ \frac{1}{\gamma _s M} &{} (\gamma _s \ll \gamma _i) \end{array}\right. } , \end{aligned}$$

where $\gamma _{s(i)} = \pi \Delta f^{s(i)}_{1/2}$, and $\Delta f^{s(i)}_{1/2}$ represents the FWHM of the signal (idler) photons. The derivation is provided in the Supplementary Information.

## Supplementary Information

Supplementary Information.

## Data Availability

The experimental data and source code supporting the findings of this study are available from the corresponding author on reasonable request.
