# Supplementary material for: Massive-mode polarization entangled biphoton frequency comb
Source: Sci Rep. 2022 May 27;12:8964. doi: 10.1038/s41598-022-12691-7 (PMC9142512; doi:10.1038/s41598-022-12691-7)
Supplement: Supplementary file 1 — Supplementary Information. [file 41598_2022_12691_MOESM1_ESM.pdf]

Supplementary Information for Massive-mode polarization entangled biphoton frequency comb

Tomohiro Yamazaki,[1] Rikizo Ikuta,[1,2] Toshiki Kobayashi,[1,2] Shigehito Miki,[3,4]
Fumihiro China,[3] Hirotaka Terai,[3] Nobuyuki Imoto,[2] and Takashi Yamamoto[1,2]

[1] *Graduate School of Engineering Science, Osaka University, Toyonaka, Osaka 560-8531, Japan*
[2] *Center for Quantum Information and Quantum Biology, Osaka University, Osaka 560-8531, Japan*
[3] *Advanced ICT Research Institute, National Institute of Information and Communications Technology (NICT), Kobe 651-2492, Japan*
[4] *Graduate School of Engineering, Kobe University, Kobe 657-0013, Japan*

(Dated: April 15, 2022)

Analysis of state generated from the singly resonant PPLN/WR and its second-order cross-correlation function

We briefly summarize the theoretical analysis of photon pair generation by SPDC inside a cavity, called the cavity-enhanced SPDC [1–8]. Then, we explain the fitting function used for the analysis of the cross-correlation function of the photon pairs and its relationship with the spectral properties of the generated BFCs. A photon pair generated by a cavity-enhanced SPDC is represented by

$$|\Psi\rangle = \int d\omega_i d\omega_s f(\omega_i, \omega_s) a^\dagger(\omega_i) a^\dagger(\omega_s) |0\rangle$$
$$f(\omega_i, \omega_s) \propto \alpha_p(\omega_i + \omega_s) h(\Delta k) \mathcal{A}(\omega_i) \mathcal{A}(\omega_s). \tag{S1}$$

$f(\omega_i, \omega_s)$ represents the joint spectral amplitude (JSA) of the signal and idler photons, normalized by $\int d\omega_i d\omega_s |f(\omega_i, \omega_s)|^2 = 1$. $\alpha_p(\omega_i + \omega_s)$ denotes the spectral amplitude of the pump light. In the CW pump regime, the linewidth of the CW pump laser is sufficiently narrow, and we approximate it to $\alpha_p(\omega_i + \omega_s) \propto \delta(\omega_p - \omega_i - \omega_s)$. $h(\Delta k) = L\mathrm{sinc}(L\Delta k/2)$ represents a function of the phase-matching condition, where $\Delta k(\omega_i, \omega_s) = n(\omega_i + \omega_s)(\omega_i + \omega_s) - n(\omega_i)\omega_i - n(\omega_s)\omega_s$, L denotes the length of the nonlinear crystal, and n denotes the refractive index[9]. $\mathcal{A}(\omega)$ is a function that represents the transmission spectrum of the cavity [10–12]; it is represented as

$$\mathcal{A}(\omega) \propto \sum_{m=0}^{\infty} \frac{\sqrt{\gamma(\omega)}}{\gamma(\omega) - i(\omega - m\Delta(\omega))} \tag{S2}$$

for a symmetric cavity without internal losses, where $\Delta(\omega) = \frac{\pi c}{n(\omega)L}$. L denotes the length of the resonator; we assume that is equal to the length of the nonlinear crystal, and c represents the speed of light. The resonant frequencies ω_m for integers m, which are the peaks of the observed spectrum, are determined by the solutions of the equation $\omega = m\Delta(\omega)$. The FWHM is $\Delta f_{1/2} = \gamma/\pi$ and the FSR is $\frac{c}{2n_g L}$, where $n_g(\lambda_0) = n(\lambda_0) - \lambda_0 \frac{dn}{d\lambda_0}$ and λ_0 represent the group index and the wavelength in vacuum, respectively.

The temperature-dependent Sellmeier equation described in Ref. [13] for the rough estimation of the refractive index $n(\lambda_0)$ is used to calculate the phase-matching function $|h(\Delta k)|^2$ shown in Fig. S1a. For the doubly resonant configuration, where both signal and idler photons are confined by the cavity, photon pair generation in most frequency ranges is suppressed because of the frequency dependency of the FSR, which is known as the "cluster effect" [14]. Figure S1c shows the simulation of the spectrum of the biphoton frequency comb in the doubly resonant configuration from equation (S1), where the cluster effect appears clearly.

We realized a singly resonant configuration using mirrors whose reflectance is frequency-dependent, as shown in Fig. S1b. Using these values of the reflectance, we simulate the spectrum of the biphoton frequency comb in our experiment, as shown in Fig. S1d. The biphoton frequency comb is generated with a certain intensity in all frequency ranges. The cluster effect does not appear if the singly resonant configuration is completely realized. However, this is not realized around the degenerate point of 1560.48 nm in our experiments. The cluster effect around the degenerate point causes a nonuniform shape in the spectrum of the biphoton frequency comb. The raw joint spectral intensities in Fig. S1c and S1d are very sensitive to the frequency difference because of their fine comb shape. Thus, we need to integrate these in the bandwidth for the measurement of 0.3 nm for comparison with the measurement results of the JSI. Figs. S1e and S1f show the results after integrating Fig. S1c and S1d, in this range, respectively.

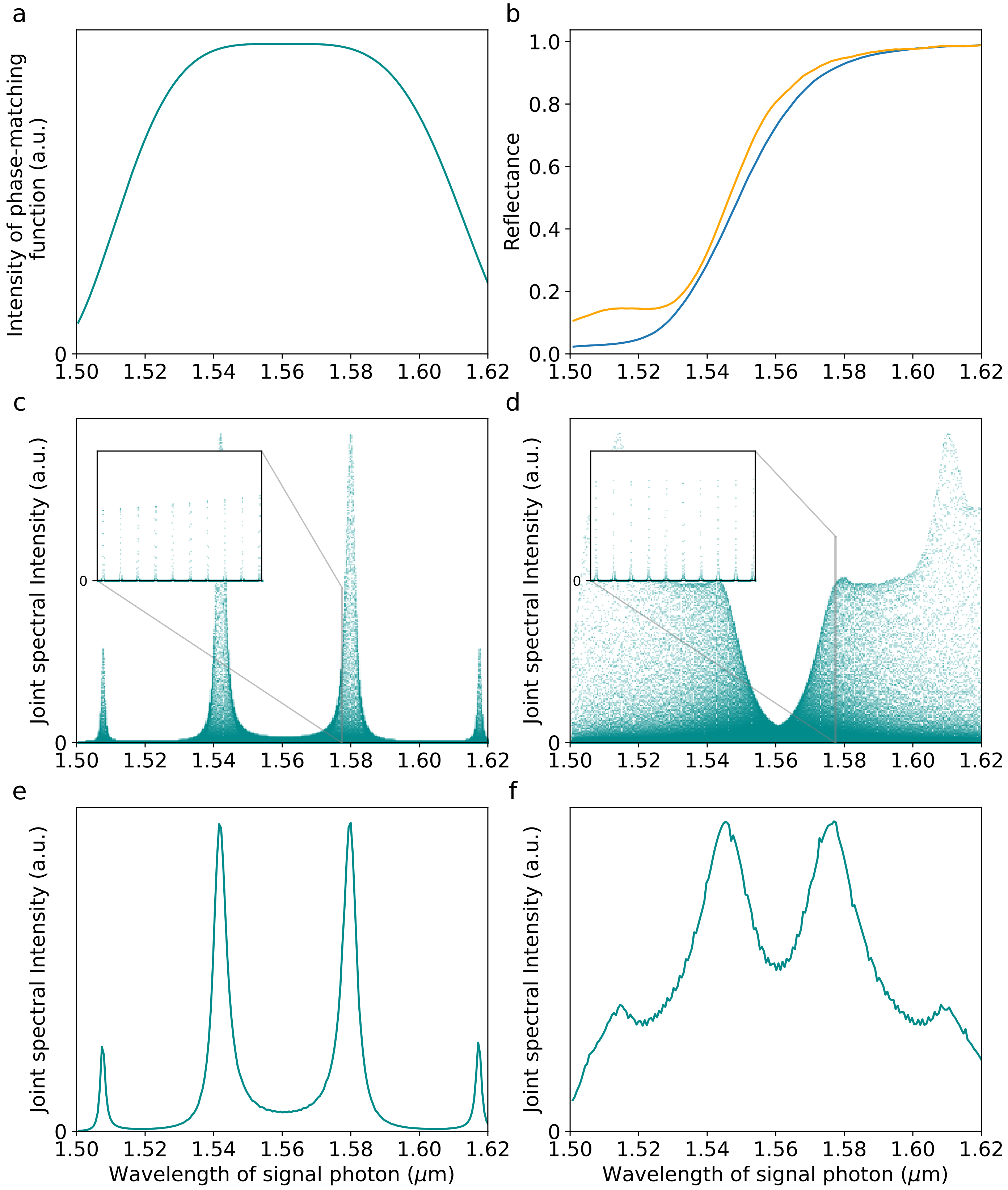

Figure S1: Simulation of the frequency spectra of biphoton frequency combs. a Phase-matching function $|h(\Delta k)|^2$. The refractive indices are calculated from Ref. [13], where we conveniently set the periodic polling period to satisfy $\Delta k = 0$ at 1560.48 nm and the temperature to $T = 69.95°\mathrm{C}$. **b** Frequency dependence of the reflectance of our waveguide resonator. The two curves represent the reflectances of the front and back-end faces. **c** Frequency spectrum of biphoton frequency comb in doubly resonant configuration. We set the reflectance $R_{r(l)} = \left|r_{r(l)}\right|^2 = 0.9$ and $\gamma = -c \log(r_r r_l)/2n(\omega)L$ and neglected the internal loss. **d** Frequency spectrum of the biphoton frequency comb in the singly resonant configuration. The insets in **b** and **c**, whose frequency ranges are [1577.30 nm, 1577.57 nm], show the periodic peaks at the resonant frequencies. **e** (**f**) Results after integrating the JSI of **c** (**d**) over a frequency range of 0.3 nm .

We further simplified JSA $f(\omega_i, \omega_s)$ to analyze the cross-correlation measurements. The frequency windows of these measurements in our experiment are up to 3 nm, which is sufficiently small to neglect the frequency dependency of $n(\omega)$ and $\gamma(\omega)$ and the cluster effect within its frequency windows. Therefore, we replace $m\Delta$ by a resonant frequency ω_m. When we set the pump frequency to satisfy $\omega_m + \omega_{m'} \simeq \omega_p$ and represent $\gamma(\omega_{s(i)})$ by $\gamma_{s(i)}$, we have

$$\begin{aligned} f(\omega_i, \omega_s) &\propto \delta(\omega_p - \omega_i - \omega_s) \sum_{m,m'} \frac{1}{\gamma_i - i(\omega_i - \omega_m)} \frac{1}{\gamma_s - i(\omega_s - \omega_{m'})} \\ &= \delta(\omega_p - \omega_i - \omega_s) \sum_{m} \frac{1}{\gamma_i - i(\omega_i - \omega_m)} \frac{1}{\gamma_s + i(\omega_i - \omega_m)}. \end{aligned} \tag{S3}$$

In the last equality, only the term about m' that satisfies $\omega_{m'} = \omega_p - \omega_m$ is left. The case $\gamma_i = \gamma_s$ corresponds to the (equally) doubly resonant configuration, and the case $\gamma_i \to \infty$ corresponds to the singly resonant configuration, where only signal photons are confined.

In the measurement of the coincidence counts between the signal and idler photons with a finite temporal resolution, the observed cross-correlation $C_{s,i}$ is described by

$$C_{s,i}^{(1,1)}(\tau) \propto \int d\tau' g_{\sqrt{2}\sigma}(\tau - \tau') G_{s,i}^{(1,1)}(\tau'), \tag{S4}$$

where

$$G_{s,i}^{(1,1)}(\tau) \equiv \langle \Psi | a_s(t)^\dagger a_i(t+\tau)^\dagger a_i(t+\tau) a_s(t) | \Psi \rangle \tag{S5}$$

is a temporal second-order cross-correlation function, and

$$g_\sigma(t) = \frac{1}{\sqrt{2\pi}\sigma} \exp\left\{ -\frac{t^2}{2\sigma^2} \right\} \tag{S6}$$

is a Gaussian function that approximates the finite temporal resolution of the two photon detectors used for coincidence measurement. [3].

From equations (S1), (S3), and (S4),

$$C_{s,i}^{(1,1)}(\tau) \propto \int d\tau' g_{\sqrt{2}\sigma}(\tau - \tau') \left| \sum_m \int d\omega_s \frac{e^{-i(\omega_p - \omega_s)\tau'}}{\{\gamma_i + i(\omega_s - \omega_m)\}\{\gamma_s - i(\omega_s - \omega_m)\}} \right|^2 \tag{S7}$$

$$\propto \int d\tau' g_{\sqrt{2}\sigma}(\tau - \tau') \left[e^{-2\gamma_i \tau'} \left| \sum_m e^{i\omega_m \tau'} \right|^2 h(\tau') + e^{2\gamma_s \tau'} \left| \sum_m e^{i\omega_m \tau'} \right|^2 h(-\tau') \right]. \tag{S8}$$

$h(\tau)$ represents the step function [7]. Equation (S8) becomes when we extract a single-frequency mode using BPFs:

$$\begin{aligned} C_{s,i,\text{single}}^{(1,1)}(\tau) &\propto \int d\tau' g_{\sqrt{2}\sigma}(\tau - \tau') \left(e^{-2\gamma_i \tau'} h(\tau') + e^{2\gamma_s \tau'} h(-\tau') \right) \\ &\propto \frac{1}{2} e^{(2\gamma_i\sigma)^2 - 2\gamma_i \tau} \left\{ 1 - \text{erf}\left(-\frac{\tau}{2\sigma} + 2\gamma_i \sigma \right) \right\} + \frac{1}{2} e^{(2\gamma_s\sigma)^2 + 2\gamma_s \tau} \left\{ 1 + \text{erf}\left(-\frac{\tau}{2\sigma} - 2\gamma_s \sigma \right) \right\}, \end{aligned} \tag{S9}$$

where $\text{erf}(x)$ denotes an error function. When we extract many frequency modes enough to the approximation,

$$\left| \sum_m e^{i\omega_m \tau'} \right| = \left| \sum_m e^{2\pi i m \frac{\tau'}{T_0}} \right| \propto \sum_n \delta(\tau' - nT_0), \tag{S10}$$

where $T_0 = 2\pi/\text{FSR}$ represents the round-trip time of the cavity. Equation (S8) then becomes

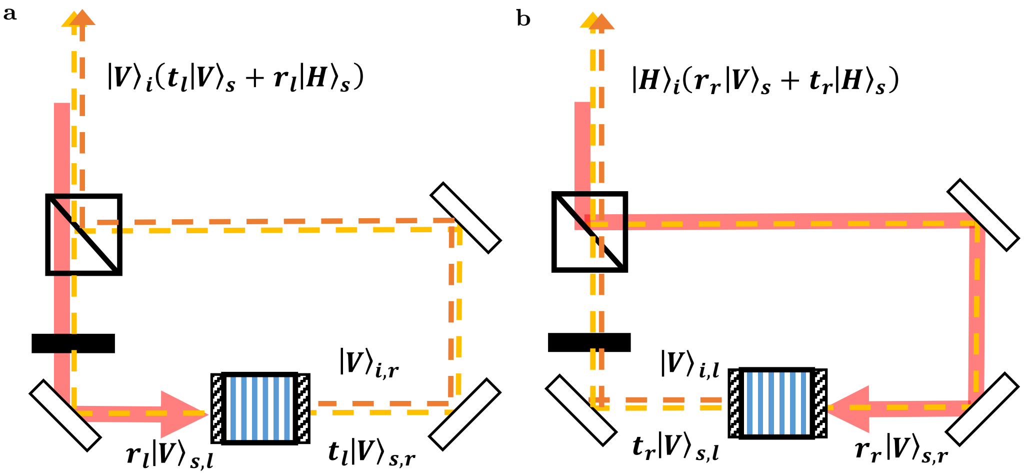

Figure S2: Sagnac interferometer with cavity-enhanced spontaneous parametric down-conversion. The thick red arrow represents the propagation of the pump laser and the thin yellow (orange) arrow represents the propagation of the signal (idler) photons. The subscript r (l) indicates the photon exiting the right (left) side of PPLN/WR.

$$C^{(1,1)}_{s,i,\mathrm{multi}}(\tau) \propto \frac{1}{2\sqrt{\pi}\sigma}\left[\exp\left(\frac{-\tau^2}{4\sigma^2}\right) + \sum_{j=1}^{\infty}\left[\exp\left(-2\gamma_i jT_0 - \frac{(jT_0-\tau)^2}{4\sigma^2}\right) + \exp\left(-2\gamma_s jT - \frac{(jT_0+\tau)^2}{4\sigma^2}\right)\right]\right]. \tag{S11}$$

Coincidence counts are represented by equation (S9) if the state is a statistical mixture of the photon pairs of the different frequency modes. The cross-correlation can be described by considering a mixture of equations (S9) and (S11) with a normalization factor with relative weight p as

$$C^{(1,1)}_{s,i,\mathrm{sum}}(\tau) \propto p\frac{(1-e^{-2\gamma_i T})(1-e^{-2\gamma_s T})}{1-e^{-2(\gamma_i+\gamma_s)T}}C^{(1,1)}_{s,i,\mathrm{multi}}(\tau) + (1-p)\frac{2\gamma_i\gamma_s}{\gamma_i+\gamma_s}C^{(1,1)}_{s,i,\mathrm{single}}(\tau). \tag{S12}$$

In our experiment, the best fit of this function in equation (S12) to the observed temporal second-order cross-correlation in Fig. S4 leads to $p \simeq 1$.

Analysis of the state generated from the Sagnac interferometer and its fidelity

We considered only one photon-pair state generated by the SPDC. As shown in Fig. S2, owing to the cavity structure for the signal photons, H-polarized and V-polarized pump lights respectively generate photon pairs described by

$$g_H e^{i(\omega_p\tau_l+\theta(\omega_p))}\left(t_l\,|V\rangle_{i,r}\,|V\rangle_{s,r} + r_l\,|V\rangle_{i,r}\,|V\rangle_{s,l}\right), \tag{S13}$$

$$g_V e^{i\omega_p\tau_r}\left(t_r\,|V\rangle_{i,l}\,|V\rangle_{s,l} + r_r\,|V\rangle_{i,l}\,|V\rangle_{s,r}\right), \tag{S14}$$

where $g_{H(V)}$, $\theta(\omega)$, and $\tau_{l(r)}$ represent the coupling constant proportional to the complex amplitude of the H (V)-polarized pump light, frequency-dependent phase from a multiorder HWP, and propagation time of the left (right) arm of the Sagnac interferometer in Fig. S2, respectively. $t_{l(r)}$ and $r_{l(r)}$ represent the transmittance and reflectance of the PPLN/WR when the light enters from the left (right) side, respectively, where $\left|t_{l(r)}\right|^2 + \left|r_{l(r)}\right|^2 = 1$ holds. The output state just before photon detection is represented by

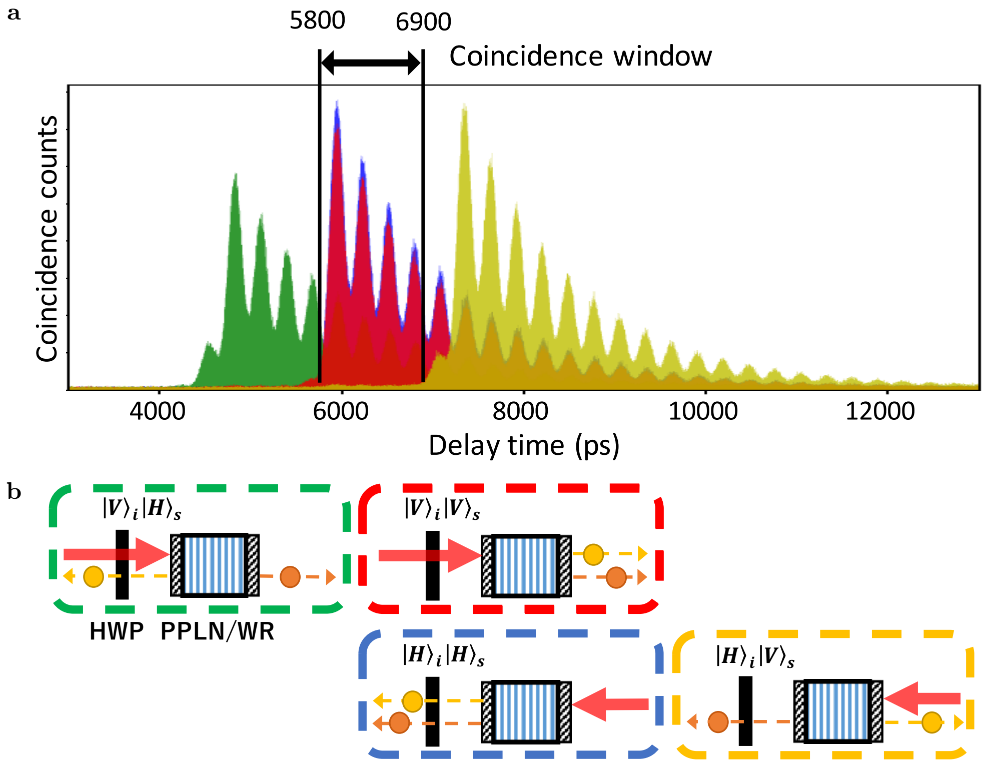

Figure S3: Correspondence of the arrival time, path, and polarization of the four output states. a Experimental data of coincidence counts in four different polarization settings, HH (blue), VV (red), VH (green), and HV (yellow), at wavelengths of 1580.48 nm and 1540.98 nm. VH (HV) photon pairs are detected earlier (later) than the VV and HH photon pairs. **b** The paths of the polarizing photons in the Sagnac interferometer (see Fig. S2). The difference in the arrival times is attributed to the difference in propagation paths.

$$\begin{aligned}
|\Psi\rangle \propto\ & g_H \eta_{ir} e^{i(\omega_p \tau_l + \theta(\omega_p) + \omega_i \tau_r)} \left(t_l \eta_{sr} e^{i(\omega_s \tau_r)} |V\rangle_i |V\rangle_s + r_l \eta_{sl} e^{i(\omega_s \tau_l + \theta(\omega_s))} |V\rangle_i |H\rangle_s \right) \\
& + g_V \eta_{il} e^{i(\omega_p \tau_r + \omega_i \tau_l + \theta(\omega_i))} \left(t_r \eta_{sl} e^{i(\omega_s \tau_l + \theta(\omega_s))} |H\rangle_i |H\rangle_s + r_r \eta_{sr} e^{i\omega_s \tau_r} |H\rangle_i |V\rangle_s \right) \\
& \propto g_H \eta_{ir} |V\rangle_i \left(t_l \eta_{sr} |V\rangle_s + r_l \eta_{sl} e^{-i\Delta\theta} |H\rangle_s \right) + g_V \eta_{il} e^{i\phi} |H\rangle_i \left(t_r \eta_{sl} |H\rangle_s + r_r \eta_{sr} e^{i\Delta\theta} |V\rangle_s \right), \qquad \text{(S15)} \\
& \phi = \theta(\omega_s) + \theta(\omega_i) - \theta(\omega_p), \quad \Delta\tau = \tau_r - \tau_l, \quad \Delta\theta = \omega_s \Delta\tau - \theta(\omega_s), \qquad \text{(S16)}
\end{aligned}$$

where $\eta_{i(j)l(r)}$ represents the total loss in the optical circuit for the signal (idler) photons coming from the left (right) side of the PPLN/WR. By setting the polarization of the pump light to satisfy $g_H \eta_{ir} t_l \eta_{sr} = g_V \eta_{il} t_r \eta_{sl}$, we obtain

$$|\Psi\rangle \propto |V\rangle_i \left(|V\rangle_s + \beta_H e^{-i\Delta\theta} |H\rangle_s \right) + e^{i\phi} |H\rangle_i \left(|H\rangle_s + \beta_V e^{i\Delta\theta} |V\rangle_s \right), \qquad \text{(S17)}$$

where

$$\beta_H \equiv \frac{r_l \eta_{sl}}{t_l \eta_{sr}}, \qquad \beta_V \equiv \frac{r_r \eta_{sr}}{t_r \eta_{sl}}. \qquad \text{(S18)}$$

For $\Delta\tau \gg 0$, we obtain the maximally polarization-entangled state $|\Phi\rangle \equiv 1/\sqrt{2}\left(|H\rangle_i |H\rangle_s + e^{i\phi} |V\rangle_i |V\rangle_s\right)$. However, in our experiment, there is an overlap between $|V\rangle_i |H\rangle_s$ and $|\Phi\rangle$, as indicated in Fig. S3. To estimate these effects on the fidelity, we consider the delay time dependency of the generated state as

$$\begin{aligned}
|\Psi(\tau)\rangle \propto\ & |V, t\rangle_i \left(e^{-\gamma\tau} h(\tau) |V, t+\tau\rangle_s + \beta_H e^{-i\Delta\theta} e^{-\gamma(\tau+\Delta\tau)} h(\tau+\Delta\tau) |H, t+\tau\rangle_s \right) \\
& + e^{i\phi} |H, t\rangle_i \left(e^{-\gamma\tau} h(\tau) |H, t+\tau\rangle_s + \beta_V e^{i\Delta\theta} e^{-\gamma(\tau-\Delta\tau)} h(\tau-\Delta\tau) |V, t+\tau\rangle_s \right). \qquad \text{(S19)}
\end{aligned}$$

Here, we approximated the temporal shaping of the state by $e^{-\gamma\tau}$. In our experiment, we selected the time windows from 0 to $\Delta\tau$, i.e.,

$$\left|\overline{\Psi}\right\rangle \equiv \int_0^{\Delta\tau} d\tau \left|\Psi(\tau)\right\rangle \propto \left|\Phi\right\rangle + \frac{\beta_H}{\sqrt{2}} e^{-\gamma\Delta\tau - i\Delta\theta} \left|V\right\rangle_i \left|H\right\rangle_s . \tag{S20}$$

We redefined this as

$$\left|H(V)\right\rangle_i \left|H(V)\right\rangle_s \equiv \int_0^{\Delta\tau} e^{-\gamma\tau} \left|H(V), t\right\rangle_i \left|H(V), t+\tau\right\rangle_s . \tag{S21}$$

Further, the randomization of the phase of the component $\left|V\right\rangle_i \left|H\right\rangle_s$ leads to

$$\rho_{\overline{\Psi}} \propto \left(\left|\Phi\right\rangle \left\langle\Phi\right| + \frac{\left|\beta_H\right|^2}{2} e^{-2\gamma\Delta\tau} \left|VH\right\rangle \left\langle VH\right|_{is} \right). \tag{S22}$$

From the definition of fidelity, $F(\rho) \equiv \max_\theta \left\langle\Psi_\theta\right|\rho\left|\Psi_\theta\right\rangle$, where $\left|\Psi_\theta\right\rangle \equiv 1/\sqrt{2}\big(\left|HH\right\rangle + e^{i\theta}\left|VV\right\rangle\big)$,

$$F(\rho_{\overline{\Psi}}) = \left(1 + \frac{\left|\beta_H\right|^2}{2} e^{-2\gamma\Delta\tau}\right)^{-1} \tag{S23}$$

holds. From these results, we estimated the fidelity expected in the case of $\Delta\tau \gg 0$ by multiplying $F(\rho_{\overline{\Psi}})^{-1}$ with the experimentally measured fidelity (Fig. 5 in the manuscript). From the best fit to the data of the temporal cross-correlation in every polarization and frequency range, we extracted γ and β_H, which are estimated as the ratio of the amplitude between the VH and HH components.

Analysis of the generated polarization entangled BFC

In the manuscript, we discuss a simplified model for generating polarization-entangled BFCs. Here, we analyze the polarization-entangled BFCs generated in the experiment using a more realistic model. As well as equation (S1), the time evolution of the cavity-enhanced SPDC for arbitrary pump power is represented by

$$\left|\Psi\right\rangle \propto \exp\left[g_{H(V)} \int d\omega_i d\omega_s f_{H(V)}(\omega_i, \omega_s) a^\dagger_{H(V)}(\omega_i) a^\dagger_{H(V)}(\omega_s) - h.c.\right] \left|0\right\rangle \equiv U_{H(V)} \left|0\right\rangle . \tag{S24}$$

Equation (S24) is reduced to approximately equation (S1) when $\left|g\right|^2 \ll 1$ is satisfied. In the CW pump regime, the JSA $f_{H(V)}(\omega_i, \omega_s)$ cannot be decomposed like $\sum_m c_{H(V),m} f_{H(V),m}(\omega_i) f'_{H(V),m}(\omega_s)$. Instead, we decompose JSA as

$$f_{H(V)}(\omega_i, \omega_s) \propto \delta(\omega_p - \omega_i - \omega_s) \sum_m c_{H(V),m} f_{H(V),m}(\omega_i). \tag{S25}$$

$c_{H(V),m}$ represents the coefficient of each cavity mode and $f_{H(V),m}(\omega_i)$ is normalized by $\int d\omega_i \left|f_{H(V),m}(\omega_i)\right|^2 = 1$. We assume that each frequency mode is well separated from the others, that is, $\int d\omega_i f^*_{H(V),m}(\omega_i) f_{H(V),m'}(\omega_i) \simeq \delta_{m,m'}$. The generation processes of each cavity mode are independent.

$$U_{H(V)} \left|0\right\rangle \simeq \prod_m \exp\left[g_{H(V)} c_{H(V),m} \int d\omega_i f_{H(V),m}(\omega_i) a^\dagger_{H(V)}(\omega_i) a^\dagger_{H(V)}(\omega_p - \omega_i) - h.c.\right] \left|0\right\rangle \equiv \prod_m U_{H(V),m} \left|0\right\rangle . \tag{S26}$$

Modifying equation (S17) for an arbitrary pump power using the above results, the state generated by our experiments can be represented as

$$\left|\Psi\right\rangle = \prod_m U_{H,m} U_{V,m} \left|0\right\rangle , \tag{S27}$$

where we ignored the component of the reflected signal photons. In the case of $|g_H c_{H,m}|^2 + |g_V c_{V,m}|^2 \ll 1$, equation (S27) is approximately

$$|\Psi\rangle \simeq \bigotimes_m \frac{1}{\sqrt{1+|g_H c_{H,m}|^2+|g_V c_{V,m}|^2}}$$
$$\left(1 + g_H c_{H,m}\int d\omega_i f_{H,m}(\omega_i) a_H^\dagger(\omega_i) a_H^\dagger(\omega_p-\omega_i). + g_V c_{V,m}\int d\omega_i f_{V,m}(\omega_i) a_V^\dagger(\omega_i) a_V^\dagger(\omega_p-\omega_i)\right)|0\rangle_m\,. \tag{S28}$$

This state can be considered a frequency-multiplexed entangled photon pair, which is similar to the state in equation (3) in the manuscript. When $\sum_m(|g_H c_{H,m}|^2 + |g_V c_{V,m}|^2) \ll 1$, the state is approximately

$$|\Psi\rangle \simeq \frac{1}{\sqrt{1+\sum_m(|g_H c_{H,m}|^2+|g_V c_{V,m}|^2)}}$$
$$\left(1+\sum_m\left(g_H c_{H,m}\int d\omega_i f_{H,m}(\omega_i) a_H^\dagger(\omega_i) a_H^\dagger(\omega_p-\omega_i), + g_V c_{V,m}\int d\omega_i f_{V,m}(\omega_i) a_V^\dagger(\omega_i) a_V^\dagger(\omega_p-\omega_i)\right)\right)|0\rangle\,. \tag{S29}$$

After removing the vacuum state, this state may correspond to that in equation (2) in the manuscript. The difference between these states discussed here and the simplified model in the manuscript is that the spectral correlation between the signal and idler photons remains in each mode in these states; this often has unfavorable effects on the interference between independent photon pairs.

We have to use a pulsed-pump regime to generate the exact frequency-bin entangled state, which is expressed as $M^{-1/2}\sum_{m=1}^{M}|\omega_{i,m}\rangle|\omega_{s,m}\rangle$. This is approximately attained in the doubly resonant configuration by making the FWHMs of the cavities for the signal and idler photons considerably smaller than the FWHM of the pump laser because the frequencies of the signal and idler photons are determined independently and almost exclusively by the FWHMs of the cavities, respectively. In the singly resonant configuration, the exact frequency-bin entangled state itis also approximately attained by making the FWHM of the cavity for the signal photon considerably smaller than the FWHM of the pump laser. Then, the frequencies of the signal and idler photons are determined almost exclusively by the FWHMs of the cavity and pump laser, respectively. In this case, the FWHMs of both photons would differ significantly from each other.

Theory of the autocorrelation function and the cavity mode in CW pump regime

Ref.[15] reported that a value $g^{(2)}$ relevant to $g^{(2)}(\tau)$ of the signal or idler photon of a photon pair in the pulse pump regime is related to the number of effective modes of a photon pair. The effective mode indicates the Schmidt mode of continuous variable Schmidt decomposition [16, 17]. Under the assumption of a uniform spectral amplitude, the relationship between $g^{(2)}$ and the number of effective modes N is expressed as $g^{(2)} = 1 + \frac{1}{N}$. A similar discussion is held in the CW pump regime [7], wherein $g^{(2)}(0) \simeq 1 + \frac{1}{M}$ is derived with the assumption that detectors have a finite temporal resolution, where M represents the number of cavity modes. These results yield the same formula and have been applied in many experiments using pulse lasers [18, 19] and CW lasers laser [20–22]. Here, we present another formula for the CW pump regime, which is independent of the temporal resolution of the detectors.

From equation (S3), we define $f(\omega_i)$ and $f_m(\omega_i)$ as

$$f(\omega_i,\omega_s) \equiv \delta(\omega_p-\omega_i-\omega_s) f(\omega_i) \equiv \delta(\omega_p-\omega_i-\omega_s)\frac{1}{\sqrt{M}}\sum_{m=1}^{M} f_m(\omega_i), \tag{S30}$$

$f(\omega_i)$ and $f_m(\omega_i)$ are normalized. $f_m(\omega_i)$ represents the normalized spectral amplitude of each cavity mode, and we assume that each cavity mode is orthogonal. We calculate the second-order autocorrelation function of the idler photons of the photon pairs as

$$G_{i,i}^{(2)}(\tau) = \langle\Psi|a_i(t)^\dagger a_i(t+\tau)^\dagger a_i(t+\tau) a_i(t)|\Psi\rangle\,. \tag{S31}$$

Using the second-order term in equation (S24) without polarization dependency as the lowest-order approximation for $|\Psi\rangle$, we obtain

$$
\begin{aligned}
&G^{(2)}_{i,i}(\tau) \\
&\propto \left\| a_i(t+\tau)a_i(t)\int d\omega_{i1}\int d\omega_{i2} f(\omega_{i1})f(\omega_{i2})a_i^\dagger(\omega_{i1})a_s^\dagger(\omega_p-\omega_{i1})a_i^\dagger(\omega_{i2})a_s^\dagger(\omega_p-\omega_{i2})\left|0\right\rangle \right\|^2 \\
&= \left\| \frac{1}{2\pi}\int d\omega_{i1}\int d\omega_{i2} f(\omega_{i1})f(\omega_{i2})\left(e^{i\omega_{i1}\tau}+e^{i\omega_{i2}\tau}\right)a_s^\dagger(\omega_p-\omega_{i1})a_s^\dagger(\omega_p-\omega_{i2})\left|0\right\rangle \right\|^2 \\
&= \frac{2}{(2\pi)^2}\int d\omega_{i1}\int d\omega_{i2}|f(\omega_{i1})|^2|f(\omega_{i2})|^2\left(2+e^{i(\omega_{i1}-\omega_{i2})\tau}+e^{-i(\omega_{i1}-\omega_{i2})\tau}\right) \\
&= \frac{1}{\pi^2}\left[\left|\int d\omega_i|f(\omega_i)|^2\right|^2+\left|\int d\omega_i|f(\omega_i)|^2e^{-i\omega_i\tau}\right|^2\right] \\
&= \frac{1}{\pi^2}\left[1+2\pi\left|\mathcal{F}\left[|f(\omega_i)|^2\right](\tau)\right|^2\right].
\end{aligned}
\tag{S32}
$$

where $\mathcal{F}\left[|f(\omega_i)|^2\right]$ represents the Fourier transform of the spectral intensity of the idler photon. When we define the normalized autocorrelation function as $g^{(2)}_{i,i}(\tau) \equiv G^{(2)}_{i,i}(\tau)/G^{(2)}_{i,i}(\tau\to\infty)$, we obtain

$$g^{(2)}_{i,i}(\tau) = 1+2\pi\left|\mathcal{F}\left[|f(\omega_i)|^2\right](\tau)\right|^2. \tag{S33}$$

This result shows that $g^{(2)}_{i,i}(0)=2$, which is independent of the spectral characteristics of photon pairs. In the single mode case [7], equation (S33) is calculated as

$$g^{(2)}_{i,i}(\tau) = 1+\left|\frac{1}{\gamma_i-\gamma_s}\left(\gamma_i e^{-\gamma_s|\tau|}-\gamma_s e^{-\gamma_i|\tau|}\right)\right|^2. \tag{S34}$$

In the multimode case, $g^{(2)}_{i,i}(\tau)$ becomes more complex, and therefore, we alternatively define

$$\Delta g^{(2)}_{i,i} \equiv \int d\tau\left(g^{(2)}_{i,i}(\tau)-1\right). \tag{S35}$$

The range of the temporal integral is assumed to be sufficiently large. From equation (S33),

$$\Delta g^{(2)}_{i,i} = 2\pi\int d\tau\left|\mathcal{F}[|f(\omega_i)|^2](\tau)\right|^2 = 2\pi\int d\omega_i|f(\omega_i)|^4. \tag{S36}$$

Substituting equation (S30) to equation (S36), we obtain

$$
\begin{aligned}
\Delta g^{(2)}_{i,i} &= \frac{2\pi}{M^2}\sum_m\int d\omega_i|f_m(\omega_i)|^4 \\
&= \frac{2\pi}{M}\left\{\int d\omega\frac{1}{\left(\gamma_i^2+\omega^2\right)^2}\frac{1}{\left(\gamma_s^2+\omega^2\right)^2}\right\}\left\{\int d\omega\frac{1}{\gamma_i^2+\omega^2}\frac{1}{\gamma_s^2+\omega^2}\right\}^{-2} \\
&= \frac{1}{M}\frac{\gamma_i^2+3\gamma_i\gamma_s+\gamma_s^2}{\gamma_i\gamma_s(\gamma_i+\gamma_s)} && \text{(S37)} \\
&= \begin{cases} \frac{5}{2\gamma_s M} & (\gamma_s=\gamma_i) \\ \frac{1}{\gamma_s M} & (\gamma_s\ll\gamma_i) \end{cases}. && \text{(S38)}
\end{aligned}
$$

Table S1: Estimated properties of the generated frequency comb. The FSRs and FWHMs of the generated frequency combs in different frequency ranges are estimated from the best fit to the temporal cross-correlation measurements. Finesse (FSR/FWHM) and Q factor (frequency/FWHM) are calculated from these values. () indicates the data of the idler photon in the doubly resonant case.

Wavelength	FSR	FWHM	Finesse	Q factor
1560 nm	3.5 GHz	516(453) MHz	7(8)	3.7(4.2) $\times 10^5$
1570 nm	3.5 GHz	243(1043) MHz	15(3)	7.9(1.8) $\times 10^5$
1580 nm	3.5 GHz	126 MHz	28	1.5 $\times 10^6$
1590 nm	3.5 GHz	85 MHz	41	2.2 $\times 10^6$
1600 nm	3.5 GHz	57 MHz	62	3.3 $\times 10^6$

Therefore, if γ_i and γ_s are known, the number of cavity modes can be estimated using this formula. In addition to equation (S4), the actual value obtained from the measurement is expressed as

$$C^{(2)}_{i,i}(\tau) \propto \int d\tau' g_{\sqrt{2}\sigma}(\tau - \tau') G^{(2)}_{i,i}(\tau'). \tag{S39}$$

After integration over time, we obtained $\Delta g^{(2)}_{i,i}$ in equation (S35) as

$$\int d\tau \Big(C^{(2)}_{i,i}(\tau) - C^{(2)}_{i,i}(\tau \to \infty) \Big) \propto \int d\tau \Big(G^{(1,1)}_{i,i}(\tau) - G^{(1,1)}_{i,i}(\tau \to \infty) \Big) \propto \Delta g^{(2)}_{i,i}. \tag{S40}$$

Therefore, $\Delta g^{(2)}_{i,i}$ can be measured without depending on the temporal resolution of the detector.

In our experiment, we estimate the value of $G^{(1,1)}_{i,i}(\tau \to \infty)$ from the best fit to the data far from the point of $\tau = 0$, as indicated by the orange lines in Fig. 4. We then calculated $\Delta g^{(2)}_{i,i}$ as 2.1 ns and 0.17 ns, respectively. Combining the values of γ_s obtained in Table. S1, we estimate the number of modes M to be 1.2 and 14.9, respectively.

Full data of time histogram of coincidence counts and estimated property of frequency comb

We measured the time histogram of coincidence counts in each frequency range by setting the central wavelength of the BPFs to (1560.48 nm, 1560.48 nm), (1570.48 nm, 1550.61 nm), (1580.48 nm, 1540.98 nm), (1590.48 nm, 1531.59 nm), and (1600.48 nm, 1522.43 nm), and by setting the bandwidths of the BPFs to 3.00 nm and 0.03 nm. Figure S4 shows all the results of the fitting obtained using equation (S12) for the bandwidth of 3.00 nm and equation (S9) for the bandwidth of 0.03 nm. The fitting results indicate that $p \simeq 1$ in equation (S12), and the results of the fitting using equation (S12) are almost the same as the results of the fitting using equation (S11). The properties of the generated frequency comb, which are estimated by the best fit to the data, are summarized in Table S1. The FWHM depends on the frequency because it is related to the frequency-dependent reflectance of PPLN/WR [23].

Another method for generating the polarization entangled state using all polarization components

From equation (S17), we can utilize the entire state as the polarization-entangled state by setting the length of the arms of the Sagnac interferometer to the same values: $\tau_r = \tau_l$ with the stabilization of the relative phase. The inner product of the two terms in equation (S17) satisfies

$$\big(\langle V|_s + \beta^*_H e^{i\Delta\theta} \langle H|_s \big) \big(|H\rangle_s + \beta_V e^{i\Delta\theta} |V\rangle_s \big) \propto r^*_l t_r \big(\eta^2_{sl} - \eta^2_{sr} \big), \tag{S41}$$

where we used the relation $r^*_l t_r + t^*_l r_r = 0$. Therefore, in the symmetric loss case, that is, $\eta_{sl} = \eta_{sr}$, the state in equation (S17) represents a maximally entangled state. However, asymmetric losses degrade orthogonality and cause a decrease in the fidelity to the maximally entangled state. The lower bound of its fidelity is

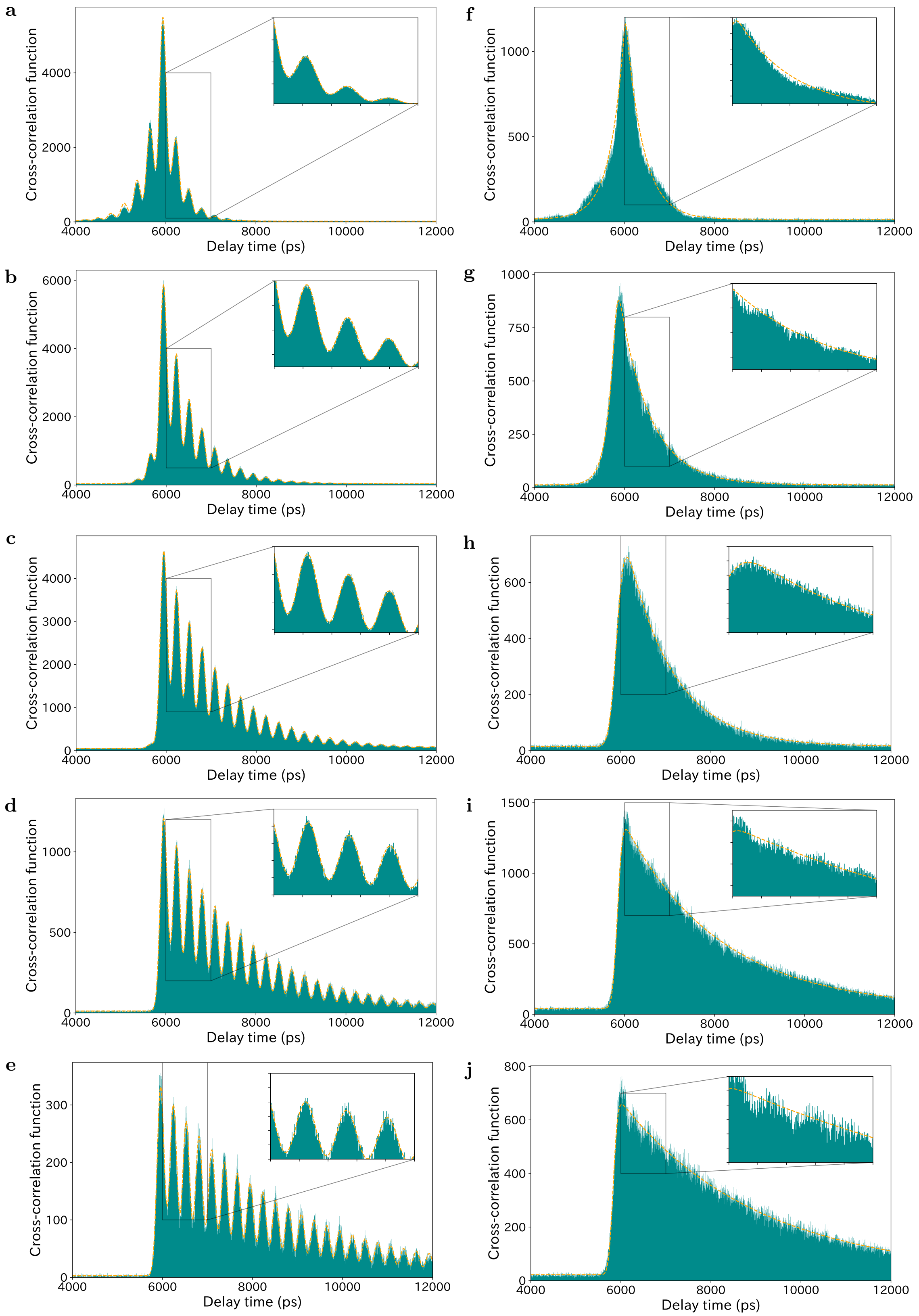

Figure S4: Full data of the temporal second-order cross-correlation. The delayed coincidence counts between the signal and idler photons with 4-ps time resolution, which correspond to the temporal second-order cross-correlation function. Their polarization is HH, but the results for the polarization of VV are almost the same as those shown in Fig. S3a. The bandwidths of the BPFs are **a-e** 3.00 nm ($\sim$ 100 frequency mode) and **f-j** 0.03 nm (single-frequency mode). The wavelengths are **a, f** (1560.48 nm, 1560.48 nm), **b, g** (1570.48 nm, 1550.61 nm), **c, h** (1580.48 nm, 1540.98 nm), **d, i** (1590.48 nm, 1531.59 nm), and **e, j** (1600.48 nm, 1522.43 nm). The data recording time is 1000 s.

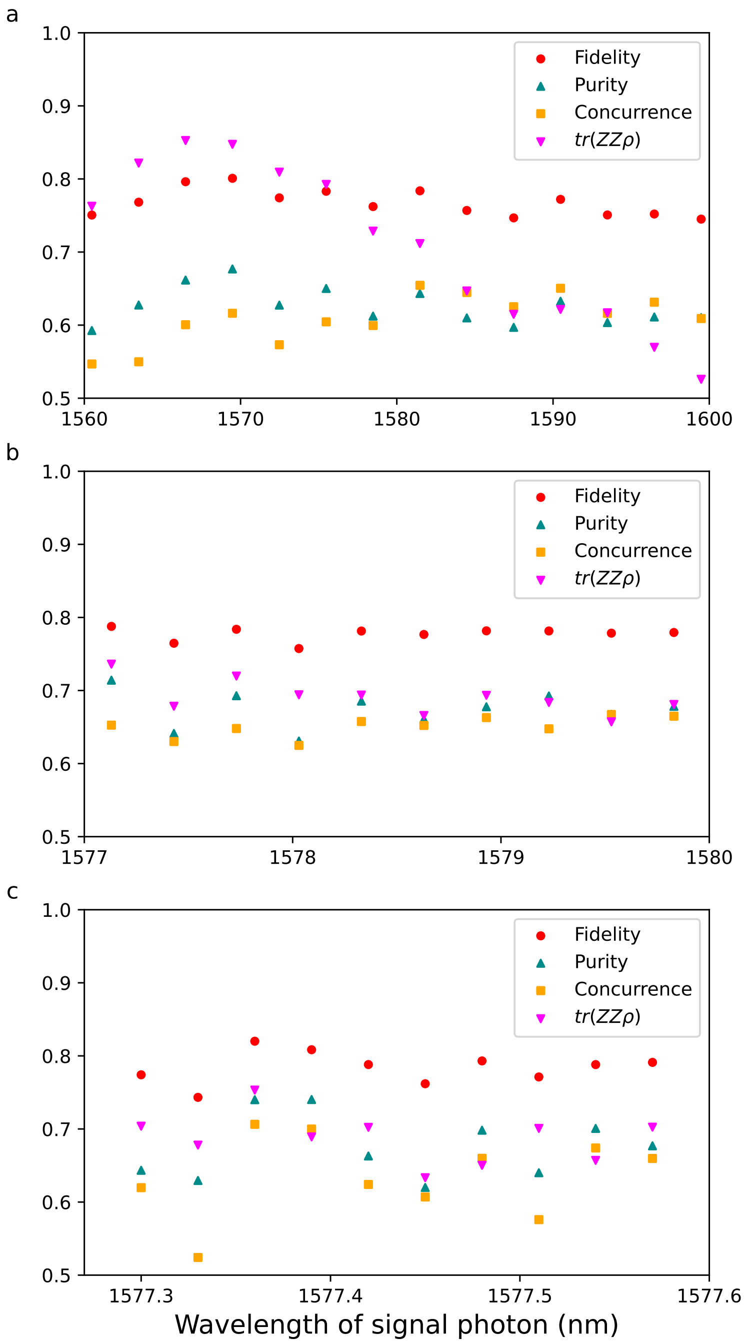

Figure S5: Characterizations of the generated entangled states. The fidelity, purity, concurrence, and $tr(ZZ\rho)$ are calculated from the reconstructed density matrices of the polarization-entangled states. The frequency ranges of a–c are the same as those shown in Fig. 5a-c in the manuscript.

$$
\begin{aligned}
F &\geq \frac{1}{2}\left[1+|1-\beta_H\beta_V| \Big/ \sqrt{\left(1+|\beta_H|^2\right)\left(1+|\beta_V|^2\right)}\right], \\
&= \frac{1}{2}\left[1+\left\{\left(1+|r|^2\left(\frac{\eta_{sl}^2}{\eta_{sr}^2}-1\right)\right)\left(1+|r|^2\left(\frac{\eta_{sr}^2}{\eta_{sl}^2}-1\right)\right)\right\}^{-1/2}\right].
\end{aligned}
\tag{S42}
$$

In the second equation, $|r_r| = |r_l| \equiv |r|$, $|t_r| = |t_l| \equiv |t|$, $|r|^2 + |t|^2 = 1$, and $1 - \beta_H\beta_V = 1 - r_r r_l / t_r t_l = |t|^{-2}$.

This lower bound is almost 1 in the typical experiments, and this method works well. This approach has twice the generation rate and does not require a temporal distinction.

REFERENCES

[1] Z. Y. Ou and Y. J. Lu, Cavity Enhanced Spontaneous Parametric Down-Conversion for the Prolongation of Correlation Time between Conjugate Photons, Physical Review Letters **83**, 2556 (1999).
[2] Y. J. Lu and Z. Y. Ou, Optical parametric oscillator far below threshold: Experiment versus theory, Physical Review A **62**, 033804 (2000).
[3] U. Herzog, M. Scholz, and O. Benson, Theory of biphoton generation in a single-resonant optical parametric oscillator far below threshold, Physical Review A **77**, 023826 (2008).
[4] M. Scholz, L. Koch, and O. Benson, Analytical treatment of spectral properties and signal-idler intensity correlations for a double-resonant optical parametric oscillator far below threshold, Optics Communications **282**, 3518 (2009).
[5] Y. Jeronimo-Moreno, S. Rodriguez-Benavides, and A. B. U'Ren, Theory of cavity-enhanced spontaneous parametric down-conversion, Laser Physics **20**, 1221 (2010).
[6] J. A. Zielińska and M. W. Mitchell, Theory of high gain cavity-enhanced spontaneous parametric down-conversion, Physical Review A - Atomic, Molecular, and Optical Physics **90**, 1 (2014).
[7] K.-H. Luo, H. Herrmann, S. Krapick, B. Brecht, R. Ricken, V. Quiring, H. Suche, W. Sohler, and C. Silberhorn, Direct generation of genuine single-longitudinal-mode narrowband photon pairs, New Journal of Physics **17**, 073039 (2015).
[8] O. Slattery, L. Ma, K. Zong, and X. Tang, Background and Review of Cavity-Enhanced Spontaneous Parametric Down-Conversion, Journal of Research of the National Institute of Standards and Technology **124**, 124019 (2019).
[9] Z.-Y. J. Ou, *Multi-photon quantum interference*, Vol. 43 (Springer, 2007).
[10] C. W. Gardiner and M. J. Collett, Input and output in damped quantum systems: Quantum stochastic differential equations and the master equation, Physical Review A **31**, 3761 (1985).
[11] D. F. Walls and G. J. Milburn, *Quantum optics* (Springer Science & Business Media, 2007).
[12] J. Garrison and R. Chiao, *Quantum optics* (Oxford University Press, 2008).
[13] D. H. Jundt, Temperature-dependent Sellmeier equation for the index of refraction, n_e, in congruent lithium niobate, Optics Letters **22**, 1553 (1997).
[14] E. Pomarico, B. Sanguinetti, C. I. Osorio, H. Herrmann, and R. T. Thew, Engineering integrated pure narrow-band photon sources, New Journal of Physics **14**, 033008 (2012).
[15] A. Christ, K. Laiho, A. Eckstein, K. N. Cassemiro, and C. Silberhorn, Probing multimode squeezing with correlation functions, New Journal of Physics **13**, 033027 (2011).
[16] S. Parker, S. Bose, and M. B. Plenio, Entanglement quantification and purification in continuous-variable systems, Physical Review A **61**, 032305 (2000).
[17] C. K. Law, I. A. Walmsley, and J. H. Eberly, Continuous Frequency Entanglement: Effective Finite Hilbert Space and Entropy Control, Physical Review Letters **84**, 5304 (2000).
[18] G. Harder, V. Ansari, B. Brecht, T. Dirmeier, C. Marquardt, and C. Silberhorn, An optimized photon pair source for quantum circuits, Optics Express **21**, 13975 (2013).
[19] M. Kues, C. Reimer, P. Roztocki, L. R. Cortés, S. Sciara, B. Wetzel, Y. Zhang, A. Cino, S. T. Chu, B. E. Little, D. J. Moss, L. Caspani, J. Azaña, and R. Morandotti, On-chip generation of high-dimensional entangled quantum states and their coherent control, Nature **546**, 622 (2017).
[20] M. Förtsch, J. U. Fürst, C. Wittmann, D. Strekalov, A. Aiello, M. V. Chekhova, C. Silberhorn, G. Leuchs, and C. Marquardt, A versatile source of single photons for quantum information processing, Nature Communications **4**, 6 (2013).
[21] X. Shi, K. Guo, J. B. Christensen, M. A. Castaneda, X. Liu, H. Ou, and K. Rottwitt, Multichannel Photon-Pair Generation with Strong and Uniform Spectral Correlation in a Silicon Microring Resonator, Physical Review Applied **12**, 1 (2019).
[22] A. Seri, D. Lago-rivera, A. Lenhard, G. Corrielli, R. Osellame, M. Mazzera, and H. D. Riedmatten, Quantum Storage of Frequency-Multiplexed Heralded Single Photons, Physical Review Letters **123**, 80502 (2019).
[23] R. Ikuta, R. Tani, M. Ishizaki, S. Miki, M. Yabuno, H. Terai, N. Imoto, and T. Yamamoto, Frequency-Multiplexed Photon Pairs Over 1000 Modes from a Quadratic Nonlinear Optical Waveguide Resonator with a Singly Resonant Configuration, Physical Review Letters **123**, 193603 (2019).
